# Study of the Humoral Immune Response towards HCV Genotype 4 Using a Bead-Based Multiplex Serological Assay

**DOI:** 10.3390/ht6040015

**Published:** 2017-10-30

**Authors:** Angela Filomena, Jens C. Göpfert, Darragh Duffy, Stanislas Pol, Mohamed Abdel-Hamid, Gamal Esmat, Arnaud Fontanet, Matthew L. Albert, Thomas O. Joos, Nicole Schneiderhan-Marra

**Affiliations:** 1 NMI Natural and Medical Sciences Institute at the University of Tuebingen, 72770 Reutlingen, Germany; angela.filomena@gmx.de (A.F.); jens.goepfert@nmi.de (J.C.G.); thomas.joos@nmi.de (T.O.J.); 2 Immunobiology of Dendritic Cells, Institute Pasteur, 75015 Paris, France; darragh.duffy@pasteur.fr (D.D.); stanislas.pol@pasteur.fr (S.P.); matthew.albert@pasteur.fr (M.L.A.); 3 Inserm U1223, Institute Pasteur, 75015 Paris, France; 4 Centre for Translational Research, Institute Pasteur, 75015 Paris, France; 5 Unité d’hépatologie, Groupe Hospitalier Cochin Hôtel-Dieu, Université René Descartes, 75006 Paris, France; 6 Department of Microbiology, Faculty of Medicine, Minia University, Minia 11432, Egypt; vhrl@link.net; 7 Endemic Medicine and Hepatogastroenterology, Faculty of Medicine, Cairo University, Cairo 12613, Egypt; gesmat@gamalesmat.com; 8 Emerging Disease Epidemiology Unit, Institute Pasteur, 75015 Paris, France; arnaud.fontanet@pasteur.fr; 9 Conservatoire National des Arts et Métiers, Unité PACRI, 75003 Paris, France

**Keywords:** hepatitis C virus genotype 4, HCV, multiplex serological assay, antibody response, acute or chronic hepatitis C virus infection

## Abstract

Hepatitis C is one of the leading causes of hepatocellular carcinoma and remains at a high prevalence in Egypt and other resource-limited countries. Several hepatitis C virus (HCV) genotypes are distributed throughout the world, with genotype 4 being most common in North and Central Africa. We developed a multiplex serological assay for the detection of the HCV specific humoral immune response, with a focus on genotype 4. For the multiplex HCV assay we used twelve antigenic regions of different HCV proteins (core, and non-structural (NS) proteins NS3, NS4, NS5A, NS5B) and validated the assay technically and clinically. In comparison to a commercially available test, our assay revealed a higher sensitivity for genotype 4, and is therefore more suited for studying immune seroconversion in samples from acutely infected Egyptian HCV patients. Furthermore, our assay discriminates acutely and chronically infected HCV patients. Of 296 well characterized HCV patient samples, 83.9% of the acute samples and 86.5% of the chronic samples could be correctly classified. In sum, this newly developed serological HCV assay has a higher sensitivity for HCV genotype 4, and can thus improve diagnostic accuracy. Through the discrimination of acutely and chronically infected HCV patients the assay may be useful in supporting clinical management of HCV patients.

## 1. Introduction

Hepatitis C is caused by the hepatitis C virus (HCV) and is a leading cause of death and morbidity [1] with high prevalence in regions such as Egypt and other Mediterranean countries. The different HCV genotypes are distributed throughout the world. Whereas subtypes 1a, 1b, 2a, and 3a are widespread over the globe, HCV genotype 4 is most common in North and Central Africa, as well as in the Middle East, where it is responsible for more than 65% of all the HCV infections [2]. Egypt has one of the highest HCV seroprevalence (≈15%) and the highest prevalence of HCV genotype 4 (>90%) [3]. Through travel to, as well as migration from, North and Central African and Middle-Eastern countries the HCV genotype 4 has also become a perceived public health concern in Europe. 

From a clinical perspective, the first six months after infection with HCV are designated as the acute phase of disease [4,5]. From one to two weeks after infection the HCV RNA can be detected in the serum [6,7] and specific anti-HCV antibodies arise after 7–8 weeks in average, with the seroconversion times ranging from 2–26 weeks [8]. The time between the infection and the positive antibody test result is referred to as the “window period” and presents a challenge for blood banks operating in regions of high HCV prevalence. It has to be mentioned that nowadays many blood banks can overcome this problem by performing reverse-transcriptase polymerase chain reaction (RT-PCR) for HCV RNA routinely on blood donations. 

Some patients show a spontaneous viral clearance during the acute stage of the infection, but around two-thirds of the patients progress to chronic HCV infection. Because of the potential for spontaneous clearance during the acute stage, the HCV infection status (acute or chronic) can play an important role for the decision of initiating an antiviral therapy. On one hand, it has been demonstrated that patients with acute HCV infection respond better to interferon-based therapy and treatment of acute hepatitis C reduces the risk of a chronic HCV infection [9,10,11]. On the other hand, therapy, which is expensive, lengthy, and often has strong side effects, can be postponed during the acute stage to await a potential spontaneous clearance [12,13,14]. To date no commercial diagnostic assays are available to distinguish patients with acute or chronic HCV infection to support such decisions. This topic remains relevant also in the era of oral direct acting antivirals, which are expensive and the availability is limited. 

With regard to the initiation of an antiviral therapy, it would be advantageous to make an early estimation of the probability of spontaneous viral clearance in a certain patient. The discovery of a specific anti-HCV antibody profile that could support such an estimation has potential clinical value.

For the study presented here, we developed a bead-based multiplex serological assay using the Luminex technology. The assay was validated for the study of the immune response towards HCV, with a special focus on genotype 4. With a unique set of samples originating from HCV patients with acute and chronic infection, we could develop a method for discrimination of both stages. Furthermore, the early antibody response profiles of HCV patients with and without spontaneous viral clearance were analyzed.

## 2. Materials and Methods

### 2.1. Human Sera

This study was performed using serum or plasma specimens generated in the course of the European FP7 SPHINX project. The HCV status of the patients (positive or negative) was determined by the detection of HCV RNA using RT-PCR. A total number of 307 HCV positive samples were obtained from Egyptian (*n* = 199) and French (*n* = 108) patients, whereof 141 had a chronic HCV infection, and 166 out of the 199 Egyptian samples were taken from patients acutely infected with HCV. Egyptian patients were recruited from two “fever hospitals” specialized in infectious diseases in Cairo, Egypt. Inclusion criteria were characteristic to clinical findings including fever or jaundice, as well as an elevated alanine aminotransferase (ALT) ≥ 3 times the upper limit of normal (ULN). The ULN depends on the used reference population in the laboratory and is usually between 30 and 50 IU/L. Acute HCV infection was identified by positive RT-PCR test result for HCV RNA with either negative or positive anti-HCV antibody (Ab) test (INNOTEST HCV Ab IV, Fujirebio Europe, Gent, Belgium). Patients with negative anti-HCV Ab and positive HCV RNA were considered as having acute hepatitis C cases. Patients with positive anti-HCV Ab and positive HCV RNA were only considered as acute hepatitis C cases if ALT values were >10 times ULN, and if there was a recent history of possible high-risk exposure to HCV (e.g., surgical procedure). Spontaneous clearance was defined as the loss of serum HCV RNA in the absence of treatment during the first six months of infection, two consecutive negative viral RNA tests and negative PCR results on all of the subsequent tests. Chronic infections were classified by consistent detection of HCV RNA by PCR throughout infection and follow-up observation. Antiviral treatment was offered to all acute HCV patients who had not spontaneously cleared the virus within six months after the onset of symptoms. 

A total number of 117 healthy control sera confirmed to be negative for HCV were obtained from blood banks in Cairo (*n* = 26) and from the Etablissement Français du Sang (French National Blood Service, France; *n* = 61) as well as purchased from Sera Laboratories International Ltd. (West Sussex, UK; *n* = 30).

For studying the immune response towards HCV genotype 4, samples (*n* = 107) of time courses of 26 patients with and without spontaneous viral clearance were obtained from Egypt. 

All of the protocols were reviewed and approved by the Institutional Review Board of the Ministry of Health and Population in Egypt and the National Hepatology and Tropical Medicine Research Institute (NHTMRI) ethics committee; all of the patients provided written informed consent. This study protocol conforms to the ethical guidelines of the 1975 Declaration of Helsinki. An overview of the samples is given in Table 1 including information about gender and age.

### 2.2. Hepatitis C Virus Antigens

Twenty seven antigens were used for the initial development of a bead-based multiplex anti-HCV assay. The performance of each antigen was evaluated and only the best performing antigens were chosen for the final assay set-up. The antigen number was reduced to twelve, whereof three antigens originate from one of the four HCV protein regions core, and non-structural (NS) proteins NS3, NS4, and NS5. For the core and the NS3 region antigens from HCV genotype 4a, 1a, and 1b were used. For NS4 and NS5 region two HCV genotype 4a antigens were used, respectively. A total number of 12 HCV antigens (see Table 2) were finally used for this study. Full-length and truncated coding DNA-sequences of the HCV proteins core, NS3, NS5A and NS5B of the genotype 4a were codon-optimized and generated for their recombinant expression in *Escherichia coli*. Nineteen amino acids at the C-terminus of the core sequence and 16 amino acids at the C-terminus of the NS5B sequence were truncated. The gene sequences were synthesized and cloned into pET 28a expression vectors containing a His6-tag (ATG:biosynthetics GmbH, Merzhausen, Germany). His-tag proteins of Core, NS3 and NS5A were expressed in *E. coli* strain BL21(DE3) and His-tag protein NS5B was expressed in HMS174(DE3) cells. All of the expressed proteins were affinity purified by their His-tag sequence. Furthermore, five commercially available recombinant HCV proteins were used: Core g1b (Cat # 111), NS3 g1a (Cat # 201), NS3 g1b (Cat # 207) and NS4 mosaic (Cat # 300) from DSI Srl (Saronno, Italy), and NS5 g1 (Cat # 219) from ProSpec-Tany TechnoGene Ltd. (Rehovot, Israel). All of the purchased HCV proteins carried a glutathione S-transferase (GST) tag. In addition, three HCV peptides (c22 g1a (amino acid position 10-53), 5-1-1 g4a (amino acid position 1694–1735) and c100 g4a (amino acid position 1920–1935)), each containing a cysteine and two 8-amino-3,6-dioxaoctanoic acid at the N-terminus, were customer specific synthesized (Intavis Bioanalytical Instruments AG, Tuebingen, Germany). The sequences of the peptides were generated as genotype specific and corresponded to the amino acid positions of the polyprotein as indicated in the CHIRON^®^ RIBA^®^ HCV 3.0 SIA (Chiron Corporation, Emeryville, CA, USA). The amino acid sequence of peptide c100 was identical for genotype 1a and 4a. The amino acid sequence of peptide c22 differed between genotype 1a and 4a just in one position (11Q→M) and the peptide 5-1-1 differed between genotype 1a and 4a in nine positions.

### 2.3. Antigen Immobilization

The immobilization of proteins was performed, as described previously [15]. Briefly, a magnetic particle processor (KingFisher 96, Thermo Scientific, Schwerte, Germany) was used to immobilize the proteins on paramagnetic carboxylated beads (MagPlex microspheres, Luminex Corp., Austin, TX, USA). The HCV proteins were covalently immobilized onto the beads (3.75 million MagPlex microspheres) using 1-ethyl-3-(3-dimethylaminopropyl)carbodiimide (EDC)/sulfo-*N*-hydroxysuccinimide (sulfo-NHS) chemistry and a coupling concentration of 50 µg/mL per protein in a volume of 125 µL. After immobilization, the beads were stored at 4 °C until further use.

Peptides were immobilized to a carrier protein using an amine-to-sulfhydryl crosslinker. First, the carrier protein bovine serum albumin (BSA) (coupling concentration: 100 µg/mL) was immobilized on beads using EDC/sulfo-NHS chemistry as described previously [15]. These BSA-beads were activated for 1 h at room temperature with 100 µL sulfosuccinimidyl 4-[*p*-maleimido-phenyl]butyrate (sulfo-SMPB) solution (1.5 mg/mL) + 100 µL phosphate buffered saline (PBS) + 0.01% (*v*/*v*) Triton X-100. The cysteine containing peptides were reduced with one molar equivalent tris(2-carboxyethyl)phosphine (TCEP). Therefore, 50 µL cysteine peptide solution (1 mM) was incubated with 50 µL TCEP solution (1 mM in PBS) for 20 min at room temperature. At the end of the reaction 150 µL PBS + 0.0125% (*v*/*v*) Triton X-100 was added. The activated beads were washed twice with 500 µL PBS + 0.005% (*v*/*v*) Triton X-100. Reduced peptides were incubated with the activated beads for 1 h at room temperature. Beads were washed twice with 500 µL PBS + 0.005% (*v*/*v*) Triton X-100, resuspended in 300 µL CBS (PBS + 1% (*w*/*v*) BSA) + 0.05% NaN_3_ and stored at 4 °C until further use.

All of the single-bead populations with immobilized HCV antigens (proteins and peptides) were combined to generate a multiplex HCV microarray. Five internal control bead populations were added to the HCV microarray: (1) beads with covalently immobilized human IgG (Sigma-Aldrich, St. Louis, MO, USA) as control for the detection system; (2) beads with goat anti-human IgG antibody (Jackson ImmunoResearch, West Grove, PA, USA) as a control for the sample addition; (3) beads with GST; (4) beads with BSA and (5) beads with *E. coli* lysate as control for unspecific binding.

### 2.4. Bead-Based Multiplex Anti-Hepatitis C Virus Assay Procedure

Serum or plasma samples were prediluted 1:40 in PVXC (PBS + 0.8% polyvinylpyrrolidone (PVP) + 0.5% polyvinyl alcohol (PVA) + 0.1% casein) and finally diluted 1:5 in sample buffer (50% (*v*/*v*) CBS + 50% (*v*/*v*) LowCross-Buffer (Candor Bioscience GmbH, Wangen, Germany) + 1 mg/mL *E. coli* lysate + 0.5 mg/mL purified GST), yielding a final 1:200 dilution of the samples. After dilution, the samples were incubated for 20 min at room temperature. The HCV-microarray was diluted in assay buffer (CBS + 0.05% Tween 20) containing approximately 20 beads/µL per single-bead population and distributed on a 96-well PCR plate (50 µL per well). The assay was performed in a semi-automated fashion using the magnetic particle processor, as mentioned above. The beads were transferred from the bead source plate to 50 µL of the diluted human serum or plasma samples and incubated for 2 h at room temperature. Unbound antibodies were removed by washing the beads twice with wash buffer (100 µL PBS + 0.05% Tween 20). To detect bound human IgGs the beads were incubated for 1 h at room temperature with 50 µL of an R-phycoerythrin (R-PE) labeled goat anti-human IgG antibody (Jackson ImmunoResearch, West Grove, PA, USA) diluted to 5 µg/mL in assay buffer. After two washing steps with 100 µL wash buffer, the beads were resuspended in 100 µL wash buffer. Measurements were performed using a Luminex FLEXMAP three-dimensional (3D) instrument operated with Luminex xPONENT software version 4.0 (Luminex Corp., Austin, TX, USA). Binding events were displayed as median fluorescence intensities (MFI) based on ≥50 measured beads per bead population. Every sample had its own sample specific background. The sample specific background was the mean of the MFI values on the BSA bead and the *E. coli* lysate bead, which was subtracted from the MFI values on the antigen carrying beads of the corresponding sample.

### 2.5. Technical Assay Validation

The bead-based multiplex anti-HCV assay was technically validated with respect to assay precision and sample stability. Assay precision is indicated as the percentage coefficient of variation (% CV). For determination of the intra-assay precision, 20 replicates of a patient sample were measured on one assay plate. For calculation of the inter-assay precision triplicates of a sample were measured on four experimental days. The determination of the precision was performed for the blank, a negative sample and three positive samples. For the assessment of the sample stability, short-term temperature stability experiments were performed and the effect of additional freeze/thaw cycles on the test result was examined. Samples were incubated at room temperature or 4 °C for 2, 4, or 24 h prior to analysis and two, three, or five additional freeze/thaw cycles were added. The determination of the sample stability was performed for one HCV negative sample and three HCV positive samples.

### 2.6. Cut-off Definition and Methods for Sample Classification

For cutoff calculations, 117 HCV negative control sera were used. Outliers were identified using the interquartile range (IQR). Values were considered to be outliers when the value was below Q1 − 1.5× IQR or above Q3 + 1.5× IQR. Outliers were excluded from the cut-off calculations. The cut-off value for every antigen (shown in Table 2) was defined as the mean MFI value + 3× standard deviation. Dividing the antigen-specific MFI value of the samples by the antigen-specific cutoff yielded the signal-to-cut-off (S/CO) ratios. Antigens with a S/CO ratio > 1 were considered to be reactive. 

To classify samples into “negative” or “positive” the S/CO values of six HCV antigens (3 core and 3 NS3 antigens) were used in combination with the following three developed rules: (1) a sample having a minimum of four reactive antigens was classified as positive; (2) a sample having exactly three reactive antigens and a sum of the NS3 S/CO ratios of minimum 12.5 was classified as positive; (3) if neither rule 1 nor rule 2 was true, a sample was classified as negative.

To classify HCV positive samples into “acute” or “chronic” six HCV antigens (3 core and 3 NS3 antigens) were used. First, the sum of the S/CO ratios (sum signal) of the three core and three NS3 antigens was calculated for each individual sample. Classification was done according to the following three developed rules: (1) a sample having a sum signal < 1527 was classified as acute; (2) a sample having a sum signal > 1527 and an ALT activity > 500 IU/L was classified as acute; (3) a sample having a sum signal > 1527 and an ALT activity < 500 IU/L was classified as chronic. If the ALT value of a sample was not available, ALT activity < 500 IU/L was supposed. 

### 2.7. Statistical Analysis

To evaluate the significance of differences between two distributions, the nonparametric Mann–Whitney *U* test was used (*p*-values). In a first step of the method development for the classification into negative/positive or acute/chronic HCV infection, a random forest algorithm was used to get an impression of the diagnostic ability of the assay. Statistical analyses were performed using the software R (RStudio V 0.97, Boston, MA, USA) and the software WEKA version 3.6 (The University of Waikato, Hamilton, New Zealand). Heat maps and hierarchical cluster analyses were generated with the software MeV Multi Experiment Viewer version 4.9 (Dana-Farber Cancer Institute, Boston, MA, USA).

## 3. Results

### 3.1. Technical and Clinical Assay Validation

The technical validation of the bead-based multiplex anti-HCV assay revealed a mean intra-assay precision of CV = 3.7 ± 2.4% and a mean inter-assay precision of CV = 4.7 ± 3.5% across all 12 antigens and all samples. No effect of up to five additional freeze/thaw cycles on the measured sample values was observed (recovery 103.3 ± 5.0%) and the mean recovery of the short-term temperature stability was 98.5 ± 7.1%. Thus, the validation results indicate a stable test system. 

**Figure 1:**
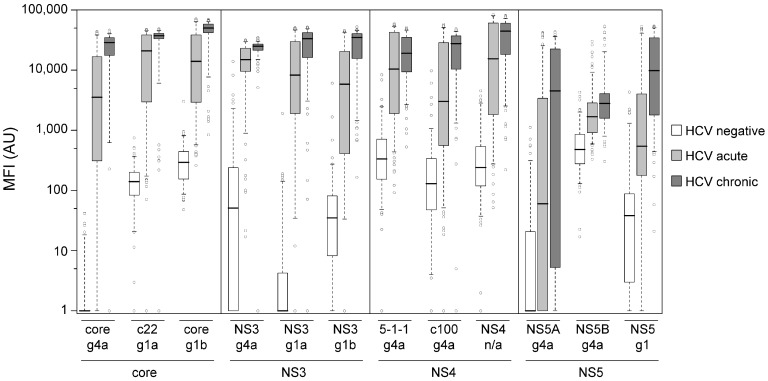
Boxplots of median fluorescence intensities (MFI) values generated with the bead-based multiplex anti-HCV assay. Every box represents the interquartile range and the horizontal line inside the box is the median. Whiskers show the fifth and the 95th percentiles. Outliers are plotted as circles. For every antigen the MFI values of HCV negative samples (*n* = 117) are shown in white boxes, samples of patients acutely infected with HCV (*n* = 166) are shown in light gray boxes and samples of patients chronically infected with HCV (*n* = 141) are shown in dark gray boxes. AU: Arbitrary units.

For the clinical assay validation, a panel of 424 sera was used, originating from patients with a defined clinical HCV status. The sample set included 307 HCV-positive and 117 HCV-negative samples. Out of the HCV-positive samples, 141 derived from chronically infected patients and 166 samples derived from acutely infected patients. The distribution of antibody reactivity towards the 12 HCV antigens is shown as boxplots in Figure 1. The reactivity of HCV negative and HCV positive samples were significantly different for all 12 antigens (see *p*-values in Table 2). Nevertheless, the HCV positive samples (acute + chronic) showed on the core and NS3 antigens a more distinct separation from the HCV negative samples than on the NS4 or NS5 antigens (Figure 1). The best diagnostic accuracy was observed for NS3 g1a (94.6%, see Table 2), but the specificity (88.9%) was not satisfying. We concluded that a classification of the samples into negative or positive for HCV based on a single antigen is not applicable and better results for sensitivity and specificity can be achieved with a combination of antigens. In the first instance for the development of such a method for classification of samples into negative or positive a random forest algorithm was used. No difference was observed for the classification result when all 12 HCV antigens or only the three NS3 and the three core antigens were used (data not shown). A subset of 225 samples (training set), consisting of 78 healthy donors, 84 patients with acute HCV infection, and 63 patients with chronic HCV infection, was used for detailed analysis of the reactivity against the NS3 and core antigens. As depicted in Table 3, all of the samples from chronic HCV patients and the most of the samples of acute HCV patients showed a minimum of three antigens with a S/CO > 1. 92.3% of the samples of healthy donors showed none or one antigen with a S/CO > 1. Therefore, it was assumed that a sample having a minimum of four reactive antigens can be classified as positive. Furthermore, if two or less antigens show a S/CO > 1 the sample is classified as negative. Positive classification of chronic HCV patient samples was possible with this first rule, but there was no clear threshold between the negative and acute HCV status. Therefore, samples of healthy donors with one or more reactive antigens were again analyzed in more detail. Reactivity against NS3 antigens was more common than reactivity against core antigens (data not shown). It was concluded that a more exact definition of a threshold as negative or positive for HCV infection is possible by using the NS3 antigens. To calculate the NS3-threshold, all the samples of healthy donors of the training set with one or more reactive antigens were used. First the sum of the S/CO values of reactive NS3 antigens was generated and the mean and standard deviation were calculated. The NS3-threshold was then defined by the mean + standard deviation with a value of 12.5. Out of this, the rule for classification of samples with exactly three reactive antigens was established: a sample having exactly three reactive antigens and a sum of the NS3 S/CO ratios of minimum 12.5 was classified as positive.

The described method for sample classification was applied to the training set (225 samples), a test set (199 samples) and the complete sample set (424 samples), and the results are shown in Table 4. All the samples of HCV-negative donors were classified correctly, which means an assay specificity of 100%. Likewise, all of the samples of patients with chronic HCV infection were classified correctly, but some of the samples of patients with acute HCV infection (11 out of 166, 6.6%) did not show any serological response to the applied antigens, for which reason these samples could not be classified as positive. The sensitivity for the samples of patients with chronic HCV infection was 100% and 93.4% for the samples of patients with acute HCV infection. In total, the assay sensitivity was 96.4%. As shown in Table 2, no single antigen could achieve such a high specificity and sensitivity. For comparison, a subset of 116 HCV-negative samples and 252 HCV-positive samples (118 acute samples and 134 chronic samples) was also measured with the commercially available INNOTEST HCV Ab IV test (see Table 5). The INNOTEST HCV Ab IV test also yielded a specificity of 100%, but a slightly lower sensitivity of 95.2%. When considering the samples of patients with acute HCV infection only, which were measured in the bead-based anti-HCV assay and the INNOTEST HCV Ab IV test, the sensitivity was found to be 92.4% with our bead-based anti-HCV assay and 89.8% with the INNOTEST HCV Ab IV test. Altogether, the bead-based multiplex anti-HCV assay showed classification results at least as good as a commercially available screening test with a slightly higher sensitivity for correct classification of samples derived from patients acutely infected with HCV genotype 4.

### 3.2. Classification of Hepatitis C Virus Patients into Acute or Chronic Stage of Infection

The HCV infection status (acute or chronic) can play an important role for the decision of initiating an antiviral therapy. Therefore, we developed a method that can classify HCV-positive samples with unknown infection status into acute or chronic stage of infection. A subset of 139 samples was used for the development of the classification method. In the first instance, a random forest algorithm was used again. There was no difference in the classification result when all 12 HCV antigens or only the three NS3 and three core antigens were used. It was concluded that there was no impact of the NS4 and NS5 reactivity towards classification into acute or chronic stage of infection. For the classification of a HCV positive sample into “acute” or “chronic” the sum of the S/CO ratios (sum signal) of the core and NS3 antigens was calculated and compared to a threshold. For the determination of the threshold, the mean and the standard deviation of the sum signal were calculated in the subset of acute samples (*n* = 75) and the chronic samples (*n* = 64), respectively. The standard deviation of the sum signal in the acute subset was subtracted from the mean (msd-a) and the standard deviation of the sum signal in the chronic subset was added to the mean (msd-c). The mean of the msd-a and the msd-c value was set as the threshold between acute and chronic HCV infection. The samples having a sum signal lower than the threshold were classified as acute HCV infection. If a sample had a sum signal higher than the threshold, the ALT activity was considered. Samples with an ALT activity > 500 IU/L were classified as acute HCV infection and samples with an ALT activity < 500 IU/L were classified as chronic HCV infection. The ALT activity threshold is based on the clinical classification for acute HCV infection during patient recruitment, thus patients with positive anti-HCV Ab and positive HCV RNA were only considered as acute hepatitis C cases if ALT values were >10 times ULN. Due to the fact that the ULN of ALT activity is dependent on the reference population [16], 500 IU/L was set as 10 times ULN. The determined threshold between acute and chronic HCV infection had to be adjusted for any new microarray production batch. The threshold for a new batch was determined correctly, with a deviation of 4.6% using 15 samples having a sum signal near the threshold. The developed method for classification of HCV positive samples into acute or chronic stage of infection was applied to the full set of 296 HCV positive samples (see Table 6). 83.9% of the acute samples and 86.5% of the chronic samples were correctly classified. In total, 85.1% of the HCV positive samples were correctly classified. A random forest algorithm, as a retrospective method, was applied to calculate the maximal possible discrimination. Using the antibody response profiles and the ALT activity values of the samples in the random forest algorithm, yielded 89.8% correctly classified samples. In sum, our method developed for classification of HCV patients into acute or chronic stage of infection showed comparable results to the retrospective random forest algorithm, and can classify HCV patients with unknown infection status into acute or chronic stage of infection.

### 3.3. Immune Response towards Hepatitis C Virus Genotype 4

**Figure A1:**
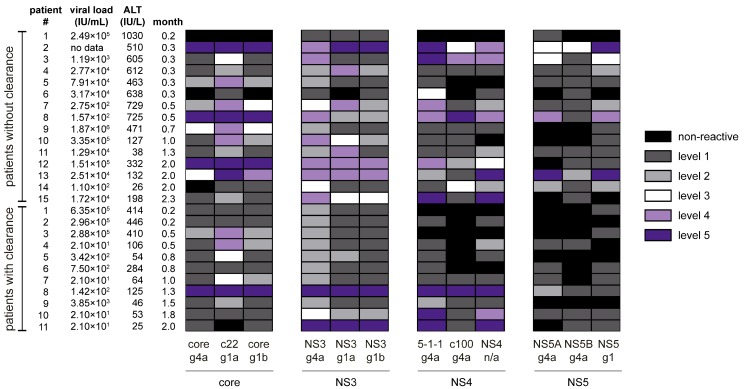
Heat map of S/CO values generated with the bead-based multiplex anti-HCV assay. For better comparability and to overcome the problem of very diverse maximal S/CO values of the antigens, the S/CO values are depicted as “non-reactive” in black (S/CO value < 1) or in colors as indicated for five different levels (five indicates highest reactivity). The S/CO levels for all antigens are shown in Table A1. Each illustrated sample is the first sample of a time course. Time courses were obtained from 15 patients without and 11 patients with spontaneous viral clearance. The samples were taken between 0.2 and 2.3 months after onset of clinical symptoms. Viral clearance was reached at a later time point.

To study the time course of the immune response towards HCV genotype 4, samples of 26 patients were obtained from the ANRS sponsored clinical study performed in Cairo, Egypt, whereof eleven patients reached a spontaneous viral clearance. Initial immune profiles (before spontaneous viral clearance) varied for individual patients (see Figure A1 and corresponding Table A1). Some of the patients showed high reactivity towards both core and NS3 antigens, whereas other patients showed a higher reactivity towards either core or NS3 antigens. Generally, the peptide c22 g1a was the most reactive antigen of the core region, and the NS3 g4a recombinant protein was the most reactive antigen of the NS3 region. While two out of the 26 samples were non-reactive on the c22 g1a antigen (92.3% reactive samples), all of the samples showed reactivity towards NS3 g4a. The peptide 5-1-1 g4a was the most reactive antigen of the NS4 region (88.5% reactive samples). Additionally, this peptide was synthesized based on the sequence of HCV genotype 1a and the reactivity of both 5-1-1 peptide versions was compared. In 65.4% of the cases, the reactivity was almost equal and for 23.1% of the samples the reactivity was higher on the 5-1-1 g4a peptide. In general, the reactivity towards NS5 antigens was less common. The most reactive antigen of the NS5 region was NS5 g1 with 73.1% reactive samples and S/CO values were in lower levels as indicated in Figure A1.

In general, the initial immune profiles of HCV patients were very heterogeneous. Comparing the immune profiles of patients with and without spontaneous HCV clearance both groups showed similar profiles. Neither a hierarchical cluster analysis nor a multivariate analysis (random forest algorithm) of an additional larger sample set (47 patients with and 43 patients without spontaneous HCV clearance) showed a possibility to differentiate between patients with and without spontaneous viral clearance. We conclude that prediction of patients who will undergo spontaneous viral clearance, is not possible based on the initial antibody response.

Analysis of samples from patients before and after spontaneous clearance indicated that the antibody titers decline within short time after elimination of the virus. In total eight of eleven patients with a spontaneous clearance (72.7%) showed a decrease of the reactivity against almost all antigens (see Table 7).

**Figure 2:**
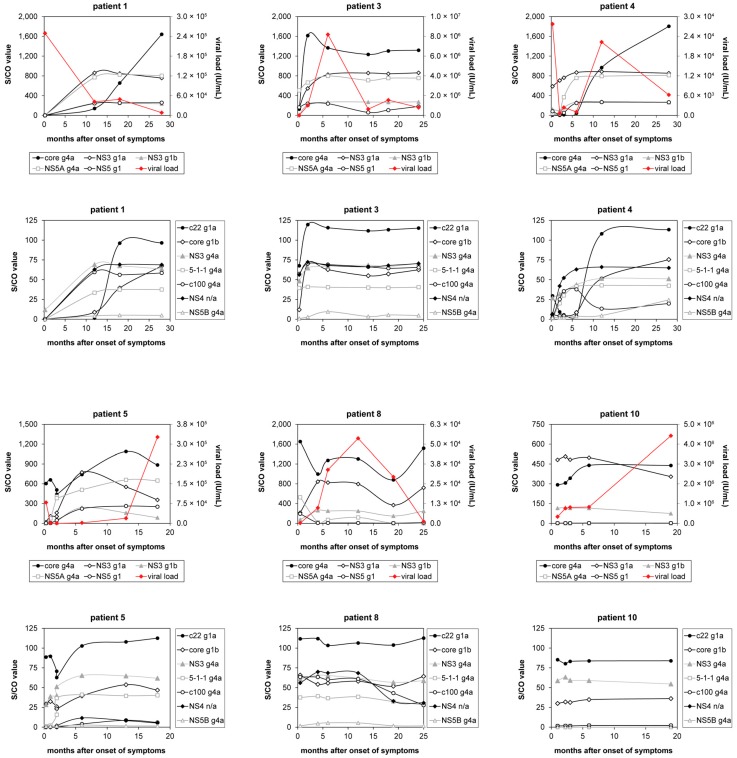
Time courses of antibody reactivity and viral load from acute to chronic HCV infection. Antibody reactivity is depicted for six exemplary patients from the data set, who developed a chronic HCV infection shown in Figure A1. For every patient two graphs were generated of which the first shows the reactivity towards five HCV antigens and the viral load. The second graph shows the reactivity towards the other seven HCV antigens.

In the time courses of the patients, who developed a chronic HCV infection, very diverse immune profiles were observed. The antibody response of six exemplary patients with differing viral load curves over time are shown in Figure 2. Some patients showed antibody response curves, which resulted in a plateau (e.g., NS4 n/a in patient 4), others showed courses, in which the reactivity remained constant over time (e.g., core g1b in patient 10). Furthermore, continuously increasing (e.g., core g4a in patient 1) and fluctuating courses (e.g., core g4a in patient 8) were observed. In some cases the antibody response increased to a certain point, and then decreased again (e.g., NS3 g1a in patient 5). In another case, the initial reactivity towards NS5A g4a in patient 8 was interestingly completely lost after 12 months. In total, the time courses of reactivity against different antigens were very heterogeneous.

## 4. Discussion

Here, we present the development and application of a method for the classification of HCV positive samples with unknown infection status into acute or chronic HCV infection. For the classification of an HCV positive sample, the sum of the S/CO ratios of the core and NS3 antigens was calculated and compared to a threshold. The technical validation of our multiplex assay showed intra- and inter-assay variations, which were significantly below the commonly accepted range of up to 15%. A comparison with the commercially available anti-HCV antibody test INNOTEST HCV Ab IV showed comparable classification results, with a slightly higher sensitivity of our bead-based anti-HCV assay for correct classification of patients acutely infected with HCV genotype 4. In contrast to the INNOTEST HCV Ab test, which uses antigens of HCV genotype 1a, 1b, 2, and 3a, we utilized antigens of HCV genotype 4a among antigens of HCV genotype 1a and 1b. However, the results of the bead-based assay indicate the importance to use optimized antigens that show equal sensitivity for all of the HCV genotypes to prevent false negative results, especially when testing samples of patients with acute HCV infection. As the anti-HCV antibody test results were compared to HCV RNA testing, a lower sensitivity in patients acutely infected with HCV was expected due to the window period before seroconversion.

In total, 85.1% of the HCV positive samples could be classified correctly as acute or chronic HCV infection. This result is only slightly lower than the maximal possible discrimination result (89.8%), which was received by using a random forest algorithm. Araujo et al. [17] have already shown the possibility for discrimination of HCV patients with acute or chronic HCV infection on the basis of the humoral immune response. They used a multivariate logistic regression model based on the antibody reactivity against six HCV antigens: one core antigen, four NS3 antigens, and one NS4 antigen. The cross-validation accuracy for their model was 90.8% for the acute samples and 97.2% for the chronic samples. Araujo et al. used samples of seroconversion panels for the group of acute HCV infection, therefore they studied only 24 different donors with acute HCV infection. In addition, the acute samples were taken no later than 62 days (two months) after the last anti-HCV-IgG-negative result [17]. We examined groups of samples from patients with acute HCV infection that were taken between 0.1 and 8.75 months after onset of symptoms. If we examine just the acute samples that were taken ≤2 months after onset of symptoms, 93.1% of the samples could be classified correctly as acute HCV infection. In summary, our developed method for the classification of HCV patients with unknown infection status into acute or chronic infection offers the possibility to supplement the clinical HCV diagnostics.

The application of our novel assay with a high specificity for HCV genotype 4 to a unique set of time course samples of patients acutely infected with HCV revealed that early antibody profiles varied greatly between patients. Some of the time course patients showed equally high reactivity towards both core and NS3 antigens, whereas some patients showed stronger reactivity towards either core or NS3 antigens. In addition, a hierarchical cluster analysis of the samples from acutely infected Egyptian HCV patients used in the clinical assay validation showed that the reactive samples can be classified into four distinct groups: (1) strong reactivity to all HCV protein regions; (2) strong reactivity to Core, NS3, and NS5 regions, but slightly weaker reactivity to the NS4 region; (3) stronger reactivity to the Core region than to the NS3 region and medium or low reactivity towards NS4 and NS5 antigens; and (4) stronger reactivity to the NS3 region than to the core region, and low or no response towards NS4 and NS5 antigens. Due to the fact that 78% of the samples from chronically infected HCV patients could be classified into the reactive groups 1 and 2 and the samples from acutely infected HCV patients were well distributed over the four reactive groups, it can be assumed that antibodies directed against NS4 and NS5 antigens arise in a later stage of seroconversion. The lack of early antibodies directed against NS4 in some of the patients can explain the lower sensitivity (87%, compare Barrera et al. [18]) of first generation anti-HCV immunoassays. Also, Xu et al. [19] observed that anti-NS5 antibodies appear relatively late, in some chronic HCV patients even after years of infection, which is in accordance with our observation.

With regard to initiation of an antiviral therapy it would be advantageous to make an early estimation of the probability for spontaneous viral clearance of individual patients. The discovery of a specific biomarker profile that could enable such a distinction would have real clinical value. However, our analysis did not yield such a specific serological immune profile that could be associated with spontaneous clearance of HCV. Therefore, identification of patients, who will undergo spontaneous viral clearance, was not possible based on the initial antibody response towards core, NS3, NS4, and NS5 antigens. To date, only antibodies towards the HCV glycoproteins E1 and E2 (not used in this study) have been described as neutralizing antibodies, which may provide a protective effect with regard to future novel vaccine candidates [20,21,22,23]. However, the high sequence variability of glycoproteins E1 and E2 makes it extremely challenging to include in a bead-based serological assay such as we have developed.

In the study of the time courses of acute HCV infection, a decrease of anti-HCV antibodies was detected after spontaneous viral clearance. However, without samples from later time points after viral eradication, it was not possible to analyze how long anti-HCV antibodies are detectable in these patients. Maylin et al. [24] and Toyoda et al. [25] showed that ten years after eradication of HCV strong reactions towards the core protein appear, whereas antibody titers towards NS3, NS4, and NS5 declined strongly.

The analysis of time courses from chronically infected HCV genotype 4 patients illustrated that the immune profiles against different HCV antigens are not static and changes in the humoral immune response can be detected even after a longer period of time.

## 5. Conclusions

In summary, the developed bead-based multiplex anti-HCV assay is a robust, specific, and sensitive approach for characterizing HCV specific antibody response. Furthermore, the assay is ideal for high-throughput serum screenings and is also perfectly suited for studying the immune response towards this neglected infectious disease [26]. Time course studies of chronic HCV genotype 4 patient samples showed that the antibody response profiles change in a dynamic manner over the course of disease. Due to its higher sensitivity for HCV genotype 4, the assay can improve diagnostic accuracy. Notably, our multiplexed serological assay discriminated acutely and chronically infected HCV patients, thereby addressing an unmet need for HCV patients. Thus, the assay may be useful in supporting clinical management of HCV patients.

## Figures and Tables

**Table 1:** Overview of samples used in this study.

HCV Status	Origin	*n* Samples	*n* Female	*n* Male	*n* Samples w/o Data	Mean Age Female	Mean Age Male
Acute	Egypt	166	61	103	2	35.0 ± 10.2	35.1 ± 10.6
Chronic	Egypt	33	18	15	-	47.8 ± 8.6	38.5 ± 12.1
Chronic	France	108	46	61	1	59.5 ± 10.1	55.1 ± 9.0
Negative	Egypt	26	n/a	n/a	26	n/a	n/a
Negative	France	61	25	36	-	35.7 ± 11.0	38.8 ± 14.2
Negative	USA	30	15	15	-	37.7 ± 13.7	45.5 ± 13.8
Time ourse * (Acute/Chronic)	Egypt	107 (26 patients)	10	16	-	34.4 ± 9.5	35.8 ± 11.7

* ANRS study. w/o: Without; n/a: Not available.

**Table 2:** Cut-off values and number of reactive samples in the bead-based multiplex anti-hepatitis C virus (HCV) assay.

HCV Protein	Antigen Name Including Genotype	Cut-off (MFI)	*p*-Value HCV Neg. vs. HCV Pos.	S/CO > 1			Diagnostic Accuracy (%)
				HCV Negative (*n* = 117) *n* (%)	HCV Acute (*n* = 166) *n* (%)	HCV Chronic (*n* = 141) *n* (%)	
core	core g4a	23	1.12 × 10^−54^	4 (3.4%)	142 (85.5%)	135 (95.7%)	92.0
	c22 g1a	399	1.97 × 10^−53^	4 (3.4%)	145 (87.4%)	136 (96.5%)	92.9
	core g1b	900	2.34 × 10^−54^	3 (2.6%)	146 (88.0%)	140 (99.3%)	94.3
NS3	NS3 g4a	442	4.14 × 10^−54^	19 (16.3%)	159 (95.8%)	140 (99.3%)	93.6
	NS3 g1a	56	2.58 × 10^−55^	13 (11.1%)	157 (94.6%)	140 (99.3%)	94.6
	NS3 g1b	165	9.77 × 10^−52^	12 (10.3%)	141 (84.9%)	140 (99.3%)	91.0
NS4	5-1-1 g4a	1258	5.65 × 10^−49^	12 (10.3%)	142 (85.5%)	138 (97.9%)	90.8
	c100 g4a	751	1.13 × 10^−44^	9 (7.7%)	116 (69.9%)	135 (95.7%)	84.7
	NS4 n/a	1129	3.05 × 10^−48^	12 (10.3%)	135 (81.3%)	138 (97.9%)	89.2
NS5	NS5A g4a	52	2.87 × 10^−45^	21 (18.0%)	85 (51.2%)	103 (73.1%)	67.0
	NS5B g4a	1631	9.24 × 10^−42^	8 (6.8%)	86 (51.8%)	102 (72.3%)	70.0
	NS5 g1	199	2.75 × 10^−46^	16 (13.7%)	119 (71.7%)	138 (97.9%)	84.4

All *p*-values are below the Bonferroni corrected significance level α’ = 0.01/12 antigens = 8.33 × 10^−4^. neg.: Negative; pos.: Positive; n/a: Genotype information not provided by supplier; S/CO: Signal to cutoff ; MFI: Median fluorescence intensity.

**Table 3:** Number of NS3 and core antigens with a S/CO > 1.

		Number of NS3 and Core Antigens with a S/CO > 1						
		0	1	2	3	4	5	6
**HCV status**	**Negative**	56	16	4	2	0	0	0
	**Acute**	3	4	3	6	8	9	51
	**Chronic**	0	0	0	0	2	3	58

**Table 4:** Diagnostic ability of the developed HCV assay.

		Training Set	Test Set	Complete Sample Set
*n* Neg. HCV		78	39	117
*n* Acute HCV		84	82	166
*n* Chronic HCV		63	78	141
**Classification**	True positive	136	160	296
	False positive	0	0	0
	False negative	11	0	11
	True negative	78	39	117
Diagnostic accuracy (%)		95.1	100	97.4
Sensitivity (%)		92.5	100	96.4
Specificity (%)		100	100	100
Positive predictive value (%)		100	100	100
Negative predictive value (%)		87.6	100	91.4

**Table 5:** Comparison of the diagnostic ability of the developed HCV assay and the commercial INNOTEST HCV Ab IV in a subset of samples.

		Multiplex HCV Assay			INNOTEST HCV Ab IV		
*n* Neg. HCV		116	116	116	116	116	116
*n* Acute HCV		118	118	0	118	118	0
*n* Chronic HCV		134	0	134	134	0	134
Classification	True positive	243	109	134	240	106	134
	False positive	0	0	0	0	0	0
	False negative	9	9	0	12	12	0
	True negative	116	116	116	116	116	116
Diagnostic accuracy (%)		97.6	96.2	100	96.7	94.9	100
Sensitivity (%)		96.6	92.4	100	95.5	89.8	100
Specificity (%)		100	100	100	100	100	100
Positive predictive value (%)		100	100	100	100	100	100
Negative predictive value (%)		92.8	92.8	100	90.6	90.6	100

**Table 6:** Classification of HCV positive samples into acute or chronic stage of infection.

		Classified as	
		Acute	Chronic
Clinical HCV status	Acute (*n* = 155)	130 (83.9%)	25 (16.1%)
	Chronic (*n* = 141)	19 (13.5%)	122 (86.5%)

**Table 7:** Change of reactivity after spontaneous viral clearance.

Patient	1	2	3	4	5	6	7	8	9	10	11
**Months after last positive HCV RT-PCR**	2.3	1.3	3.3	2.8	5.3	3.5	3.5	3.0	2.0	3.5	2.3
**Decline of antibody titer**	yes	no	no	yes	yes	no	yes	yes	yes	yes	yes
**core g4a**	32	290	135	13	62	n/r	79	74	60	73	53
**c22 g1a**	41	309	108	35	46	117	85	92	93	74	n/r
**core g1b**	35	238	127	26	53	91	82	87	78	58	94
**NS3 g4a**	56	115	118	36	83	101	82	100	91	88	101
**NS3 g1a**	39	290	123	8	62	92	52	85	62	60	100
**NS3 g1b**	33	431	85	6	71	89	50	94	60	73	100
**5-1-1 g4a**	n/r	806	270	14	91	154	80	85	99	98	82
**c100 g4a**	140	123	309	14	n/r	111	394	100	102	77	70
**NS4 n/a**	115	196	266	26	119	109	209	98	77	75	95
**NS5A g4a**	n/r	n/r	n/r	15	n/r	n/r	47	67	209	70	n/r
**NS5B g4a**	n/r	n/r	n/r	0	102	92	n/r	70	101	79	61
**NS5 g1**	63	144	176	9	n/r	111	71	43	269	46	122

The percentage (%) of the reactivity after eradication is shown based on the last S/CO value before spontaneous viral clearance. n/r (non-reactive) means that no reactivity was measured in the sample of the last positive HCV RT-PCR. RT-PCR: Reverse-transcription polymerase chain reaction.

## Appendix

**Table A1:** Levels of S/CO values.

HCV Protein	Antigen Name Including Genotype	S/CO Value, where Level Starts					Maximal S/CO Value
		1	2	3	4	5	
core	core g4a	1	404	808	1211	1614	2018
	c22 g1a	1	25	50	74	98	123
	core g1b	1	17	32	48	64	80
NS3	NS3 g4a	1	17	32	48	64	79
	NS3 g1a	1	188	374	561	747	934
	NS3 g1b	1	64	128	191	254	317
NS4	5-1-1 g4a	1	10	19	29	38	47
	c100 g4a	1	16	31	46	61	77
	NS4 n/a	1	17	34	50	66	82
NS5	NS5A g4a	1	170	340	509	678	847
	NS5B g4a	1	8	15	22	29	36
	NS5 g1	1	56	110	165	220	274

Depicted are the S/CO values, where the level starts. The levels for an antigen were defined by evenly dividing the maximal observed S/CO value into five parts. The maximal S/CO value was defined during clinical assay validation.
